# Effect of the Low Fermentable Oligosaccharides, Disaccharides, Monosaccharides, and Polyols (FODMAP) Diet on Control of Pediatric Irritable Bowel Syndrome and Quality of Life Among a Sample of Egyptian Children: A Randomized Controlled Clinical Trial

**DOI:** 10.7759/cureus.61017

**Published:** 2024-05-24

**Authors:** Sarah A El Ezaby, Ayat F Manzour, Marwa Eldeeb, Yasmin G El Gendy, Diaa M Abdel Hamid

**Affiliations:** 1 Family Medicine, Faculty of Medicine, Ain Shams University, Cairo, EGY; 2 Community, Environmental and Occupational Medicine, Faculty of Medicine, Ain Shams University, Cairo, EGY; 3 Pediatrics, Faculty of Medicine, Ain Shams University, Cairo, EGY; 4 Pediatric Nutrition, Faculty of Medicine, Ain Shams University, Cairo, EGY

**Keywords:** abdominal pain, quality of life, low fodmaps diet, dietary intervention, irritable bowel syndrome

## Abstract

Background: Irritable bowel syndrome (IBS) is a pediatric pain-dominant functional gastrointestinal disorder that has a negative impact on all children's dimensions of quality of life. A dietary approach that focuses on limiting food elements with high fermentable oligosaccharides, disaccharides, monosaccharides, and polyols (FODMAP) can be used to decrease symptoms of IBS. This study aims to evaluate the effect of low FODMAP dietary intervention on health-related quality of life among a sample of Egyptian children.

Methods: Eighty-four children aged 5-15 years old were randomly assigned to two groups, 42 patients in the low FODMAP diet group and 42 patients in the standard diet group. They received the diet for six weeks and were followed up weekly using a visual analog scale (VAS) for pain severity assessment, the Pediatric Quality of Life (PedsQL) Inventory Gastrointestinal (GI) Symptoms Module Scale, and the PedsQL Inventory Generic Core Scale to assess the physical and psychosocial functioning of the patients.

Results: The VAS score decreased more in the low FODMAP group, which caused a significant difference between the two groups (p<0.001). The PedsQL Inventory GI Symptoms Module score increased more among the low FODMAP group, and this caused a significant difference between the two groups (p<0.001). PedsQL Inventory Generic Core score increased more among the low FODMAPs group, and this caused a significant difference between the two groups (p<0.001).

Conclusion: Low FODMAP dietary intervention in pediatrics for six weeks decreased abdominal pain severity, improved gastrointestinal symptoms, and improved the health-related quality of life of the affected children.

## Introduction

Irritable bowel syndrome (IBS) is a pediatric chronic pain-dominant functional gastrointestinal disorder (FGID) with upper and lower gastrointestinal symptoms [1].

It is also one of the most common conditions encountered by both primary health care (PHC) physicians and specialists, and it accounts for approximately 12% of visits to PHC physicians and 28% of referrals to gastroenterologists [2].

IBS has a negative impact on a child’s daily activities, education, and health-related quality of life, and it can often persist into adulthood [3,4]. Most IBS patients report at least moderate pain and moderate anxiety or depression [5].

So this condition causes high levels of utilization of the healthcare system, accounts for more than 25% of all pediatric emergency consultations for abdominal pain, and leads to a significant annual healthcare cost [6,7].

According to Rome IV criteria, IBS is defined as abdominal pain that presents at least once weekly over a minimum of three consecutive months, in addition to two of the following criteria: pain related to defecation, change in stool frequency, and change in stool appearance [3].

Other symptoms that may be associated with IBS include nausea, vomiting, headaches, anorexia, and arthralgia [3]. IBS is also subdivided into four subtypes according to changes in bowel habits based on the Bristol stool form scale: IBS with diarrhea (IBS-D), IBS with constipation (IBS-C), IBS mixed type (IBS-M), and IBS un-subtyped (IBS-U) [8]. IBS pathogenesis has many theories, and food allergies are one of them [9]. The majority of IBS patients report that symptoms of exacerbation or generation are present with the intake of certain foods [3].

A recently established dietary approach involves restricting foods high in fermentable oligosaccharides, disaccharides, monosaccharides, and polyols (FODMAP) that may cause or worsen symptoms of irritable bowel syndrome (IBS) [10].

Short-chain carbohydrates known as intraluminal water ferment and create gas, dilate the intestinal lumen, and cause gastrointestinal symptoms [11].

Therefore, reducing FODMAP intake through diet can help with IBS symptoms [12].

This study aims to evaluate the effect of a low FODMAP dietary intervention on health-related quality of life among a sample of Egyptian children. Data are scarce on the application of a low FODMAP diet for managing IBS in the pediatric population within the Mediterranean region [13].

## Materials and methods

In a randomized double-arm single-blinded interventional study, we recruited participants from a general pediatric outpatient clinic in the Pediatrics' Hospital, Faculty of Medicine, Ain Shams University Hospitals, Cairo, Egypt, from October 2020 until January 2022.

The study included children aged 5-15 who were diagnosed with IBS by a pediatric gastroenterologist according to ROME IV criteria. Children were excluded when one or more of the following alarming signs were presented according to ROME IV criteria: the presence of abdominal pain or diarrhea that wakes the child from sleep, delay in onset or progression of puberty, faltering growth, a family history of inflammatory bowel disease, celiac disease, history of significant weight loss, bleeding per rectum, persistence of severe vomiting or diarrhea, persistent joint pain, recurrent unexplained fever, unexplained pallor, children with organic diseases, and known food allergies.

A sample of 38 IBS patients in each group was required to achieve a power of 0.90 to detect an effect size of 1.16 for the decrease in abdominal pain score, according to a previous study by (Dogan et al., 2020), who studied the effect of low FODMAP on IBS among children [14]. The sample was increased to 10% to reach 42 cases per group (84 total) to compensate for dropouts. A baseline assessment was done through the full history-taking and clinical examination of all participants.

Randomization

Eligible patients were sequentially randomized into two groups, the low FODMAP group and the standard diet group, for a six-week intervention period. Computer-generated random numbers were used to allocate the patients.

The time of arrival (TOA) randomization sequence was created with a random block size of four.

Parameters for evaluation

The severity of abdominal pain was assessed by a visual analog scale (VAS) at the start of the study, which starts with zero (indicates no pain) and ends with 10 (indicates the worst pain). The score is easily understood and applied by patients over the age of five [15].

The Pediatric Quality of Life (PedsQL) Inventory Gastrointestinal (GI) Symptoms Module Scale (version 3.0) (the Arabic version of the Child Report) developed by James Varni was used to ask the children and their caregivers about the frequency of GI symptoms. This scale is composed of 10 dimensions that cover the symptoms of the gastrointestinal system: stomach pain, stomach discomfort when eating, food and drink limits, trouble swallowing, reflux, nausea and vomiting, gas and bloating, constipation, blood in the stool, and diarrhea scales [16].

PedsQL Inventory Generic Core Scale (version 4.0) (the Arabic version of the Child Report) was used to assess the physical, emotional, social, and school functioning of the participants [16].

The Generic Core Scale is a multidimensional, 23-item questionnaire that measures health-related quality of life in healthy children and those with acute or chronic health conditions [16]. The dimensions are physical functioning (eight questions), emotional functioning (five questions), social functioning (five questions), and school functioning (five questions) [16].

In PedsQL questionnaires, the participants and their caregivers had to choose from the offered answers: never experienced these symptoms, almost never, sometimes, often, and almost always, with each answer given a code from 0 to 4, respectively (Table 1). To calculate the scores easily, items are reversed scored and linearly transformed to a 0-100 scale, so that higher scores indicate better health-related quality of life (HRQOL) and improvement of GI symptoms [16].

**Table 1:** PedsQL scores calculation PedsQL: Pediatric Quality of Life

Response choices	Never	Almost never	Sometimes	Often	Almost always
Raw scores	0	1	2	3	4
0-100 scale scores	100	75	50	25	0

To create the psychosocial health score, the mean is computed as the sum of the items over the number of items answered in the emotional, social, and school functioning scales. The Physical Health Summary Score is the same as the Physical Functioning Scale Score. To create the total scale score, the mean is computed as the sum of all the items over the number of items answered on all the scales. The PedsQL questionnaires are available at https://eprovide.mapi-trust.org and were used after obtaining permission.

Study procedure

In the first group (the low FODMAP group), the children and their caregivers were interviewed, and a 24-hour diet recall was recorded from the participants. After the diagnosis was done and pharmacological treatment was prescribed by the gastroenterologist, the participants and their guardians were educated about adding a dietary modification to pharmacological treatment to help improve symptoms.

A careful explanation about low FODMAP dietary intervention was offered to each child and caregiver, and a list of allowed foods (low FODMAP) and foods that should be avoided (high FODMAP) was given to each patient (see Appendices). The low FODMAP items were colored green, and the high FODMAP items were colored red. Items were colored red to give the child a visual cue to differentiate between food preferred to eat and food preferred to avoid [17]. For example, wheat- and rye-based bread were substituted by gluten- and rye-based bread, and cow's milk products were substituted by lactose-free dairy products.

Each patient was given a dietary sheet (food diary) to record the food consumed daily in the main three meals and the snacks.

To check the reliability of the answers, the child and caregiver were interviewed by each other, so any answer from one of them had to be confirmed by the other one. We emphasized that the caregiver who was interviewed on the first visit should come in for every visit. and during the week, we made sudden phone calls and asked some questions from the questionnaires to make sure of their answers.

To ensure and assess participants' adherence, participants and caregivers were requested to send what they ate or their food diary daily via SMS or WhatsApp. Unexpected calls were made during the week and a 24-hour diet recall was taken.

In the second group (the standard diet group), participants in this group were given, besides their regular treatment, careful instructions according to National Institute of Clinical Excellence (NICE), UK guidelines for IBS management, such as avoiding eating spicy food, drinking plenty of water, having regular meals, and avoiding junk food [18].

Participants in both groups were evaluated weekly for six weeks. The evaluation was done by attending the outpatient clinic, assessing gastrointestinal symptoms frequency using the PedsQL Inventory GI Symptoms Module, detecting changes in the pain analog scale, and assessing physical and psycho-social functioning in the last week using the PedsQL Inventory Generic Core questionnaire.

Ethical considerations

The study was registered on clinicaltrial.gov with ID: NCT05396651.

Approval from the Research Ethics Committee, Faculty of Medicine, Ain Shams University was taken (FMASU319/2019).

Assent was taken from children above 12 years and written consents were obtained from participants and their guardians after explaining the study design, goals, and participants' rights.

## Results

**Figure 1:**
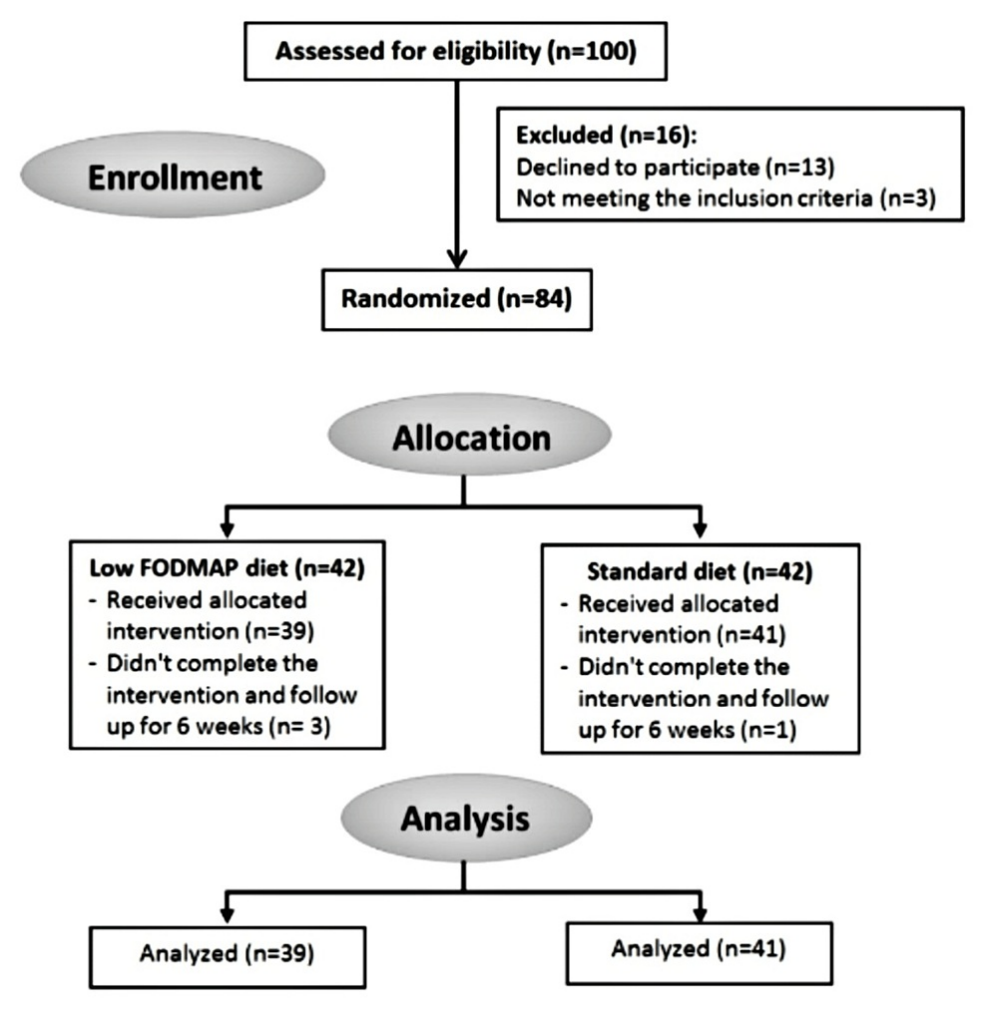
Consort flow chart. Participant flow chart, where 100 participants were examined for eligibility, 16 were excluded, and the other 84 were equally and randomly allocated in both groups (42 in each group). Thirty-nine participants completed the intervention in the low FODMAP group, and 41 completed the standard diet group. FODMAP: Fermentable oligosaccharides, disaccharides, monosaccharides, and polyols.

The present study enrolled 84 participants: 42 in the low FODMAP diet group and 42 in the standard diet group. Thirty-nine participants completed six weeks of low FODMAP intervention, and 41 participants in the standard diet group (a total of 80 participants; four participants were lost to follow-up) (Figure 1).

Most of the participants were males in both groups, with a mean age of 10.33 ±2.80 in the low FODMAP group and 10.98 ±2.59 in the standard diet group, and this mean age corresponds to child education, where most of the participating children were in the primary stage of education. There was no significant difference between the two groups in their socio-demographic characteristics (Table 2). Regarding parents' education, most of the fathers were university graduates in both groups, while most of the mothers were secondary school graduates in the low FODMAP group and university graduates in the standard diet group, with no significant difference between the two groups (Table 2).

**Table 2:** Socio-demographic characteristics of the studied participants. FE: Fisher's exact test.

		Intervention group n=39	Control group n=41	Student's t-test	P value
		Mean ±SD	Mean ±SD		
Age		10.33 ±2.80	10.98±2.59	1.06	0.29
		N (%)	N (%)	Chi-square test X^2^	P value
Gender	Male	23 (59%)	25 (61.0%)	0.03	0.86
	Female	16 (41%)	16 (39.0%)		
Child Education	Preschool	2 (5.1%)	2 (4.9%)	0.18 FE	1.00
	Primary	23 (59.0%)	25 (61.0%)		
	Preparatory	14(35.9%)	14 (34.1%)		
Paternal education	University graduate	20 (51.3%)	25 (61.0%)	1.41 FE	0.51
	Secondary education	18 (46.2%)	14 (34.1%)		
	Preparatory education	1 (2.6%)	2 (4.9%)		
Maternal education	University graduate	19 (48.7%)	22 (53.7%)	2.05 FE	0.41
	Secondary education	20 (51.3%)	17 (41.5%)		
	Preparatory education	0 (0.0%)	2 (4.9%)		

The most prevalent IBS subtype among the participating children was IBS-C in both groups, with no significant difference between the two groups regarding IBS subtypes (Table 3). There was no significant difference between the mean duration of abdominal pain between the two groups (Table 3).

**Table 3:** IBS subtypes and abdominal pain duration. IBS: Irritable bowel syndrome, IBS-C: IBS with constipation, IBS-D: IBS with diarrhea, IBS-M: IBS mixed type, IBS-U: IBS un-subtyped.

		Intervention group N=39	Control group N=41	Student's t-test	P value
		Mean ±SD	Mean ± SD		
Duration of pain in months		15±13.8	13±10.7	0.73	0.46
		N (%)	N (%)	Chi-square test X^2^	P value
IBS subtypes	IBS-C	18 (46.2%)	15 (36.6%)	3.22	0.36
	IBS-D	8 (20.5%)	11 (26.8%)		
	IBS-M	9 (23.1%)	6 (14.6%)		
	IBS-U	4 (10.3%)	9 (22%)		

Before the intervention, it was found that there was no statistically significant difference in the baseline VAS score, the GI symptoms module dimensions or the total GI symptoms score. After three weeks of intervention, it was found that there was a highly significant difference between the two groups in the VAS score and the total GI symptoms module score.

Stomach pain, gas and bloating, and diarrhea were the most prominent dimensions that showed marked improvement (Table 4). 

**Table 4:** Comparison between two groups regarding week 3 VAS score and PedsQL Inventory GI Symptoms Module score FODMAP: Fermentable oligosaccharides, disaccharides, monosaccharides, and polyols, VAS: Visual analog scale, PedsQL: Pediatric Quality of Life, GI: Gastrointestinal. * p value between the two groups after three weeks of intervention.

	Low FODMAP group n=39 Mean ± SD		Standard group n=41 Mean ±SD		T	P value*
	W0	W3	W0	W3		
VAS	9.15 ± 0.71	5.15 ± 1.23	8.90 ± 0.92	6.90 ± 0.80	7.516	<0.001
Stomach pain	44.09 ± 13.13	75.91 ± 9.72	45.80 ± 16.16	64.23 ± 14.92	4.167	<0.001
Discomfort when eating	72.05 ± 27.69	94.87 ± 10.48	74.39 ± 27.41	87.68 ± 16.32	2.356	0.021
Heartburn and reflux	72.92 ± 28.07	89.58 ± 12.93	72.56 ± 30.10	87.79 ± 15.41	0.562	0.576
Nausea and vomiting	94.39 ± 16.83	98.56 ± 5.64	96.04 ± 11.25	99.09 ± 4.09	0.481	0.632
Gas and bloating	34.88 ± 14.22	74.33 ± 12.32	36.32 ± 13.73	60.15 ± 14.80	4.643	<0.001
Constipation	60.75 ± 32.85	70.95 ± 29.42	69.18 ± 34.17	71.89 ± 32.70	0.135	0.893
Diarrhea	69.52 ± 30.73	86.25 ± 16.09	59.84 ± 32.97	71.23 ± 26.05	3.119	0.003
GI symptoms module total	71.04 ± 6.07	84.76 ± 5.05	72.34 ± 6.18	80.49 ± 6.16	3.377	0.001

The VAS score, PedsQL Inventory GI Symptoms Module, and PedsQL Inventory Generic Core scores were calculated after six weeks of dietary intervention. The mean VAS score decreased more in the low FODMAP group which caused a high significant difference between the two groups. The PedsQL Inventory GI Symptoms Module score and the PedsQL Inventory Generic Core score increased more among the low FODMAP group which caused a high significant difference between the two groups (Table 5). There was no significant difference between the two groups in the school functioning score at the beginning of the study, but after six weeks of intervention, it increased more in the low FODMAP group, which made a significant difference between the two groups (Table 5).

**Table 5:** VAS score, total GI symptoms module score, and generic core score before and after six weeks of intervention. FODMAP: Fermentable oligosaccharides, disaccharides, monosaccharides, and polyols, VAS: Visual analog scale, GI: Gastrointestinal. *P value among the low FODMAP after six weeks of intervention, † P value among the standard diet group after six weeks of intervention, ‡ P value between the two groups after six weeks of intervention.

	Low FODMAP group N=39 Mean± SD			Standard diet group N=41 Mean± SD			P ‡ value
	W0	W6	P* value	W0	W6	P † Value	
VAS	9.15 ± 0.71	0.90 ±0.79	<0.001	8.90 ± 0.92	4.54 ±1.07	<0.001	<0.001
GI symptoms module total score	71.04 ± 6.07	91.37 ±4.99	<0.001	72.34 ± 6.18	84.16 ±6.26	<0.001	<0.001
Physical	96.06 ± 5.73	98.71 ±3.42	<0.001	96.02 ± 5.46	98.69 ±3.13	<0.001	0.978
Emotional	90.13 ± 8.92	96.15 ±4.79	<0.001	90.6 ± 8.89	95.37 ±6.36	<0.001	0.535
School	72.05 ± 27.69	97.44 ±6.16	<0.001	74.39 ± 27.41	89.51 ±15.32	<0.001	0.003
Psychosocial	87.18 ± 9.94	97.82 ±2.57	<0.001	88.13 ± 9.24	94.84 ±5.01	<0.001	0.001
Total generic	90.27 ± 7.03	98.13 ±1.98	<0.001	90.87 ± 6.44	96.18 ±3.37	<0.001	0.002

Low FODMAP dietary intervention was found to independently decrease VAS scores and independently increase both PedsQL Inventory GI Symptoms Module and PedsQL Inventory Generic Core scores, with no effect of age on the improvement of scores (Table 6).

**Table 6:** Multiple regression of unit change. The independent effect of the low fermentable oligosaccharides, disaccharides, monosaccharides, and polyols (FODMAP) diet, and the patient's age on the visual analog scale (VAS), Pediatric Quality of Life (PedsQL) Inventory Gastrointestinal (GI) Symptoms Module score, and PedsQL Inventory Generic Core score.

VAS	Variables	Β	T	p-value	95.0% CI	
					Lower Bound	Upper Bound
	Intervention	-3.855	-15.024	0.000	-4.366	-3.344
	Age	0.047	0.966	0.337	-0.049	0.143
	Constant	-4.761	-8.592	0.000	-5.865	-3.657
PedsQL Inventory GI Symptoms Module score	Intervention	8.364	8.445	0.000	9.504	18.024
	Age	-0.200	-1.073	0.287	-1.383	2.654
	Constant	13.764	6.435	0.000	-0.571	0.171
PedsQL Inventory Generic Core score	Intervention	2.593	1.782	0.079	-0.559	10.077
	Age	0.053	-0.067	0.947	-2.604	2.435
	Constant	4.759	1.782	0.079	-0.411	0.516

**Figure 2:**
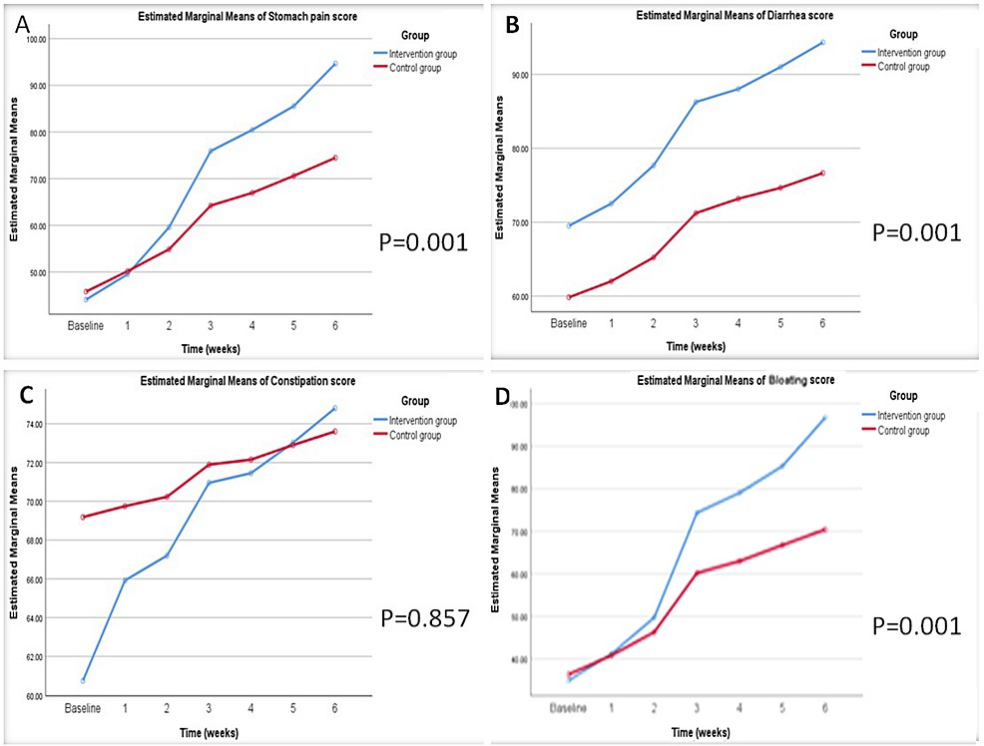
Changes in IBS symptoms throughout six weeks of intervention. IBS: Irritable bowel syndrome. A: Stomach pain, B: Diarrhea, C: Constipation, D: Bloating. Blue line for the low FODMAP diet group, and the red line for the standard diet group. P-value significant < 0.05.

Stomach pain, which is the main symptom of IBS, showed more improvement among the low FODMAP group and a high significant difference between the two groups (Figure 2A). Low FODMAP intervention showed an obvious improvement in the diarrhea score, which caused a high significant difference between the two groups (Figure 2B). A low FODMAP diet also induced an improvement in the constipation score, but there was no significant difference between the two groups (Figure 2C). Bloating, which is an associated symptom of IBS, showed marked improvement among participants in low FODMAP groups and high significant differences between the two groups (Figure 2D).

## Discussion

In the current study, the majority of participants were males (total 48 (60%)); 23 in the low FODMAP group, and 25 in the regular diet group, with a mean age of 10.33±2.8 and 10.98±2.59 in the two groups, respectively.

Castro et al. evaluated the efficacy of a low FODMAP diet in 20 children with functional abdominal pain in Spain [19]. The participants' median age was 10 (IQR, 8.25-11.75), and the gender distribution was balanced, with an equal number of males and females (10 each) [19]. Brown et al. investigated the low FODMAP diet in children and adolescents with functional bowel disorders [20]. The study included 29 children (20 females and nine males) with an average age of 11.7 ± 3.94 [20]. In Dogan et al., a study of 60 patients (30 in each group) with average ages of 13.40±2.60 and 13.46±2.75 compared low FODMAPs to the standard diet [14].

The gender distribution observed in both the aforementioned studies and the present study aligns with the reviews suggesting that there is no gender predisposition for IBS and no exact role of sex hormones on IBS [1]. Moreover, the age in the current study and the other studies corresponded to the literature review, which mentioned that the most common reported age for pediatric IBS was between 8 and 12 years [1].

The study by Dogan et al., which also assessed the pain severity by VAS score after two months of intervention, found that the mean decrease in VAS score was higher in the low FODMAP group, which agreed with the present study [14]. Castro et al. studied the effect of a low FODMAP diet on children for two weeks and found the change in VAS score was from 4.63 to 1.41 but with no statistically significant difference [19]. This finding disagreed with the results of the present study, which may be attributed to the longer duration of follow-up in the present study.

According to James Varni questionnaires, as the scores should be reversed to be calculated, high PedsQL scores indicate fewer symptoms and lower scores indicate more symptoms [16]. In the present study, after three weeks of intervention, the stomach pain and diarrhea scores increased more among the low FODMAP group. Furthermore, associated symptoms such as gas and bloating scores showed a notable increase, highlighting a significant difference between the two groups. This suggests that the low FODMAP diet effectively improved stomach pain, diarrhea, gas, and bloating, leading to an improvement in overall GI symptoms within this group.

After six weeks of intervention, the VAS score and total GI symptoms score continued to improve more among the low FODMAP group. Other dimensions like nausea, vomiting, reflux, and constipation showed more improvement in the low FODMAP group, but with no statistically significant difference. These results agreed with Brown et al., who studied the effect of low FODMAP on 29 children in New Zealand retrospectively and assessed the symptoms response to the diet two to 28 months after completion of the diet, where most of the participants reported improvement in abdominal pain, bloating, constipation, diarrhea, nausea, and reflux [20].

In the present study, the quality of life dimensions was assessed using the total PedsQL Inventory Generic Core score. It demonstrated a notable increase in scores among the low FODMAP group, with a significant difference observed between the two groups at the end of the intervention. This indicates that the low FODMAP group experienced greater improvement in symptoms and health-related quality of life compared to the standard diet group. This finding is consistent with the results of İpek et al., who conducted a pilot study in 2021 involving 10 children to evaluate the impact of a low FODMAP diet on quality of life using KINDL scores [21]. The KINDL scores assessed physical, emotional, and school well-being, as well as family and friend relationships. Their study revealed that all dimensions were affected by IBS symptoms. After four weeks of a low FODMAP diet, there was a significant increase in emotional well-being, family subscale, and total KINDL scores. Although not statistically significant, there was a positive increase in physical well-being and school subscale scores after the intervention [21].

It was noted that the most affected dimension in the quality of life score was the school functioning scale, which corresponds to Varni et al., who studied the impact of functional gastrointestinal disorders on the health-related quality of life using the PedsQL Inventory Generic Core Scale [22]. The significant impact on school functioning may indicate challenges with school policies that fail to accommodate the needs of IBS patients, such as frequent bathroom visits during the school day. Also, the negative impact of abdominal pain forced the child to be absent from school. In the present study, this dimension showed significant improvement among the low FODMAPs group.

The findings of this study show the efficacy of the low FODMAP diet as an independent factor in significantly improving VAS scores, PedsQL Inventory GI Symptoms Module, and PedsQL Inventory Life Generic Core scores among children with IBS. It also shows more improvement in GI symptoms and quality of life.

Limitations

The main limitation of our study was the high cost and the limited availability of low FODMAP dietary substitutes. So participants were offered some food items, like gluten-free and lactose-free dairy products. In addition, strict follow-up was required to ensure participants' compliance with the dietary intervention.

## Conclusions

The findings of the current study suggest that adhering to low fermentable oligosaccharides, disaccharides, monosaccharides, and polyols (FODMAP) diets leads to a significant improvement in irritable bowel syndrome (IBS) symptoms and enhances patients' quality of life within a shorter duration compared to providing general dietary advice with a standard diet. So if low FODMAP food items are available and affordable, it's better to follow this diet for controlling IBS symptoms, and if it is not, patients can follow standard guidelines, but improvement will take much more time. As patients with IBS and other functional abdominal pain disorders primarily present in primary health care settings, IBS management should be an integrated effort between general physicians, gastroenterologists, and nutritionists.

Dietary modification should be a cornerstone in the management of IBS-affected children; it improves gastrointestinal symptoms and the quality of life. Due to the higher cost of low FODMAP food items compared to other dietary options and the pressing need for increased accessibility to affordable food substitutes for IBS patients, it is imperative for the government to provide financial support for these food items and ensure their widespread availability across all governorates.

## Appendices

**Table 7:** Low and high FODMAP food items. FODMAP: Fermentable oligosaccharides, disaccharides, monosaccharides, and polyols. Table credits: Sarah A. El Ezaby

Food should be avoided	Allowed food	Food group
Wheat-, and rye-based bread and pasta/cereals/biscuits	Rice, cornflakes, oats (gluten-, rye-, and barley-free bread and pasta)	Starch: breads and cereals
Cow's milk, custard, evaporated milk, soy milk, sweetened condensed milk, yogurt	Lactose-free milk, feta cheese, hard cheeses, lactose-free yogurt	Dairy and alternatives
Most legumes, pulses, some marinated meat, poultry, seafood and processed meats	Eggs, plain cooked meat, poultry, seafood	Protein sources
Cauliflower, garlic, onions, green peas, mushrooms, broccoli, okra	Eggplants, green beans, green capsicum, carrot, cucumber, lettuce, potato, zucchini, sweet potato, spinach	Vegetables
Apples, apple juice, cherries, dried fruits, mango, nectarines, peaches, pears, plums, watermelon	Cantaloupe, kiwi, mandarin, orange, pineapple, strawberry	Fruits
High fructose corn syrup, honey	Dark chocolate, table sugar	Sugars and sweeteners
Cashews, pistachios	Peanuts	Nuts
